# Activation of endogenous angiotensin converting enzyme 2 prevents early injuries induced by hyperglycemia in rat retina

**DOI:** 10.1590/1414-431X20154583

**Published:** 2015-09-29

**Authors:** G. Foureaux, B. S. Nogueira, D. C. O. Coutinho, M. K. Raizada, J. C. Nogueira, A. J. Ferreira

**Affiliations:** 1 Departamento de Morfologia, Instituto de Ciências Biológicas, Universidade Federal de Minas Gerais, Belo Horizonte, MG, Brasil; 2 Department of Physiology and Functional Genomics, McKnight Brain Institute, University of Florida, Gainesville, FL, USA

**Keywords:** ACE2 activation, Angiotensin-(1-7), Renin-angiotensin system, Eyes, Diabetic retinopathy

## Abstract

Diabetic retinopathy (DR) is a serious complication of diabetes mellitus that may result in blindness. We evaluated the effects of activation of endogenous angiotensin converting enzyme (ACE) 2 on the early stages of DR. Rats were administered an intravenous injection of streptozotocin to induce hyperglycemia. The ACE2 activator 1-[[2-(dimethylamino) ethyl] amino]-4-(hydroxymethyl)-7-[[(4-methylphenyl) sulfonyl] oxy]-9H-xanthone 9 (XNT) was administered by daily gavage. The death of retinal ganglion cells (RGC) was evaluated in histological sections, and retinal ACE2, caspase-3, and vascular endothelial growth factor (VEGF) expressions were analyzed by immunohistochemistry. XNT treatment increased ACE2 expression in retinas of hyperglycemic (HG) rats (control: 13.81±2.71 area%; HG: 14.29±4.30 area%; HG+XNT: 26.87±1.86 area%; P<0.05). Importantly, ACE2 activation significantly increased the RCG number in comparison with HG animals (control: 553.5±14.29; HG: 530.8±10.3 cells; HG+XNT: 575.3±16.5 cells; P<0.05). This effect was accompanied by a reduction in the expression of caspase-3 in RGC of the HG+XNT group when compared with untreated HG rats (control: 18.74±1.59; HG: 38.39±3.39 area%; HG+XNT: 27.83±2.80 area%; P<0.05). Treatment with XNT did not alter the VEGF expression in HG animals (P>0.05). Altogether, these findings indicate that activation of ACE2 reduced the death of retinal ganglion cells by apoptosis in HG rats.

## Introduction

Diabetic retinopathy (DR) is one of the most frequent complications of diabetes mellitus (DM). It may be present in both type 1 and type 2 DM (1), and is a highly common cause of blindness (2). Increased incidence of DM and DR is an important concern in developing countries, and represents a significant health problem worldwide (3). Both experimental and clinical studies have shown the crucial role of sustained hyperglycemia in the pathogenesis of chronic diabetic complications. This metabolic status results in lesions in retinal small vessels, which are the most important clinical change in DR. High plasma glucose levels make the blood circulation inadequate, and activate biological systems that restore the oxygen supply to tissues through stimulation of angiogenesis (4,5).

The traditional view of the pathophysiology of DR is that damage in the microcirculation is due to the long duration of the disease. However, recent studies indicate that lesions in neuronal and glial cells may appear early in the development of DR (6,7). Therefore, the first years of DM are the most appropriate for the introduction of effective therapeutic interventions to prevent irreversible changes in the eye (8). DR treatment includes increased metabolic control, laser therapy, pharmacological approaches (antiangiogenic and anti-inflammatory therapies, enzymatic vitreolysis, and intravitreal injections), and surgery (8).

The renin-angiotensin system (RAS) is a peptidergic hormone system, which plays a central role in the pathophysiology of the eye. Different components of the RAS have been identified in the eye, such as angiotensin (Ang) II and angiotensin II type 1 receptor (AT1) (9-13). Abnormal functioning of the RAS is associated with many visual disorders, and is critically involved in the pathogenesis and progression of retinopathy induced by hyperglycemia (14,15). Evidence indicates that Ang II, acting through AT1 receptors, induces the development and progression of retinopathy by causing damages to the micro- and macrocirculation, in addition to inducing the death of neuronal and glial cells (6,7). Thus, drugs that reduce the Ang II actions might have beneficial effects on DR (16-18), although it has been reported that these drugs have limited effects in this disease (15). However, recent studies have demonstrated the existence of a novel metabolic system within the RAS composed of Ang-(1-7), angiotensin-converting enzyme (ACE) 2, and Mas receptors in the eye (9,12,19). This system acts as a counter-regulator of the ACE/Ang II/AT1 effects. Indeed, it has been found that activation of intrinsic ACE2 decreases the intraocular pressure of glaucomatous rats (19), as well as the inflammatory process observed in uveitic mice (20). Nevertheless, the role of the Ang-(1-7)/ACE2/Mas system in DR has not been fully investigated.

Cumulative evidence suggests that ACE2 activation is an innovative and efficient therapeutic strategy to treat cardiac fibrosis, pulmonary hypertension, vascular thrombosis, endothelial dysfunction, diabetic cardiomyopathy, autonomic dysfunction induced by hyperglycemia, glaucoma, and uveitis (19,21-26). Thus, in this present study, we hypothesized that activation of endogenous ACE2 might lead to improvements in the early stages of DR. To test this hypothesis, we investigated whether the compound 1-[[2-(dimethylamino) ethyl] amino]-4-(hydroxymethyl)-7-[[(4-methylphenyl) sulfonyl] oxy]-9H-xanthone 9 (XNT), an ACE2 activator, is able to modulate neuronal and vascular biomarkers of DR in hyperglycemic rats.

## Material and Methods

### Animals

Male Wistar rats (3 months of age) weighing 180-220 g were obtained from the animal facility of the Instituto de Ciências Biológicas (CEBIO, Universidade Federal de Minas Gerais, Brazil). The animals were housed in a temperature-controlled room (22-23°C) with a 12-12 h light-dark cycle. Water and food were available *ad libitum*. The experimental protocols were performed in accordance with institutional guidelines approved by the Ethics Committee in Animal Experimentation of the Universidade Federal de Minas Gerais (CETEA-UFMG), Brazil, which are in accordance with the National Institutes of Health (NIH) Guidelines for the Care and Use of Laboratory Animals (protocol #11/11). In addition, this study conformed to the Association for Research in Vision and Ophthalmology (ARVO) Statement for the Use of Animals in Ophthalmic and Vision Research.

### Diabetes induction and XNT treatment

Rats were anesthetized with an intraperitoneal injection of a mixture of ketamine (70 mg/kg) and xylazine (10 mg/kg). They were then administered with a single intravenous injection of streptozotocin (STZ; 50 mg/kg) diluted in sodium citrate buffer (10 mM, pH 4.5) to induce hyperglycemia. Non-hyperglycemic control rats (CTRL) were injected with ∼0.2 mL of sodium citrate buffer. Ten days after hyperglycemia induction with STZ, the rats were assessed for blood glucose levels, and animals with a fasting blood glucose concentration over 126 mM were considered hyperglycemic (HG) (25). After confirmation of hyperglycemia, XNT (1 mg/kg per day; HG+XNT group) or vehicle (saline pH 2-2.5; equivalent volume; HG group) was administered by daily gavage for 30 days. CTRL animals received daily gavage of saline for 30 days.

### Histological analysis

Animals were enucleated, two small sagittal sections were made in the nasal and temporal sides of the eyes, and then the eyes were immersed in Bouin’s fluid for approximately 24 h. Following the fixation, the eyes were dehydrated in different concentrations of ethanol (70, 80, 90, 95, and 100%). Diaphanization was done in xylene and the eyes were embedded in Paraplast. Serial sections with a thickness of 6 μm were obtained using a microtome (HM335E, Microm, USA). For histological analysis and counting of retinal ganglion cells (RGC), sections were stained with hematoxylin and eosin (HE). RGC were manually counted in the whole extension of the neuronal retina (n=5-6 in each group) using a microscope (BX 53, Olympus, USA).

### Immunohistochemical analysis

An immunohistochemistry technique was used to evaluate the expression of ACE2 (n=9 in each group), caspase-3 (n=5-6 in each group), and vascular endothelial growth factor (VEGF; n=9 in each group). Briefly, 6-μm-thick histological sections were diaphanized and hydrated in ethanol (100, 95, 90, 80, 70, 50, and 25%). Subsequently, peroxidase blockade was performed using 3% H_2_O_2_ for 15 min. This was followed by blockade of unspecific binding with a solution of 2% bovine serum albumin containing 0.1% Tween 20 for 1 h in a moist chamber. The primary antibodies (polyclonal rabbit anti-ACE2, 1:500, GeneTex, USA; polyclonal rabbit anti-caspase-3, 1:500, Sigma-Aldrich, USA; and polyclonal chicken anti-VEGF, 1:50, Sigma-Aldrich) diluted in the blocking solution were incubated overnight at 4°C in a humid chamber. Then, the samples were incubated with the secondary antibody for 1 h. The signal amplification was performed using a streptavidin-biotin-peroxidase kit LSAB/DAKO (Dako North America, USA) followed by incubation with 0.025% diaminobenzidine and counterstained with Harris’ hematoxylin (Merck, Germany). The sections were photographed with a 40× objective and 10 images of the retina per animal were used to quantify the expression of ACE2, caspase-3, and VEGF. The images were captured under exactly the same light. Image Pro-Plus software (Meyer Instruments, Inc., USA) was used to quantify the expression of these proteins. Positive ACE2 and VEGF expression was considered as the area occupied by brown pixels in the retina, while caspase-3 expression was analyzed using the area occupied by brown pixels only in the RGC layer.

### Statistical analysis

Data were reported as means±SE. The results were analyzed using one-way ANOVA followed by the Newman-Keuls test. All tests were performed using the GraphPad Prism 5 software (USA). The significance level was defined as P<0.05.

## Results

### Effects of XNT administration on ACE2 expression in retinas

**Figure 1:**
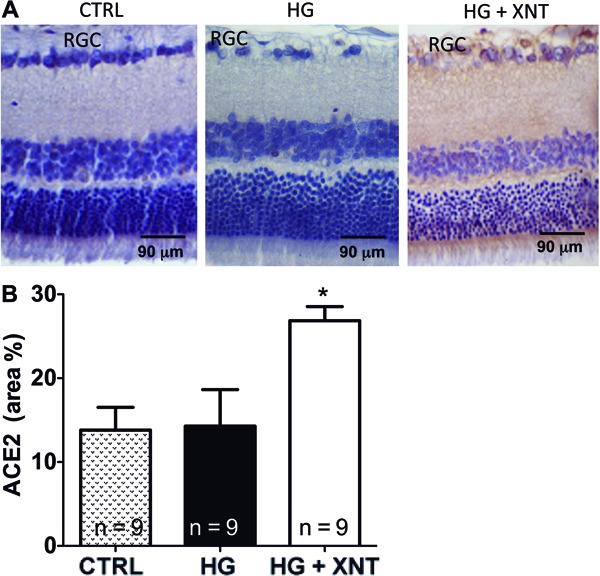
Administration of 1-[[2-(dimethylamino) ethyl] amino]-4-(hydroxymethyl)-7-[[(4-methylphenyl) sulfonyl] oxy]-9H-xanthone 9 (XNT) increased angiotensin converting enzyme 2 (ACE2) expression in retinas of hyperglycemic rats. *A*, Representative photomicrographs of retinas of control rats (CTRL), hyperglycemic untreated rats (HG), and hyperglycemic treated rats (HG+XNT). RGC: retinal ganglion cells. *B*, Quantification of ACE2 expression in retinas of rats. *P<0.05 compared to CTRL and HG groups (one-way ANOVA followed by the Newman-Keuls test).

Induction of hyperglycemia in rats did not cause any significant alteration in the expression of ACE2 in retinas (13.81±2.71 area% in the CTRL group and 14.29±4.30 area% in the HG group, P>0.05). However, daily administration of XNT for 30 days in hyperglycemic animals increased the expression of this enzyme in retinas (Figure 1A). Accordingly, quantification of this finding showed that hyperglycemic rats treated with XNT presented higher expression of ACE2 in their retinas (14.29±4.30 area% in the HG group and 26.87±1.86 area% in the HG+XNT group, P<0.05, Figure 1B).

### Effects of XNT administration on retinal ganglion cells

**Figure 2:**
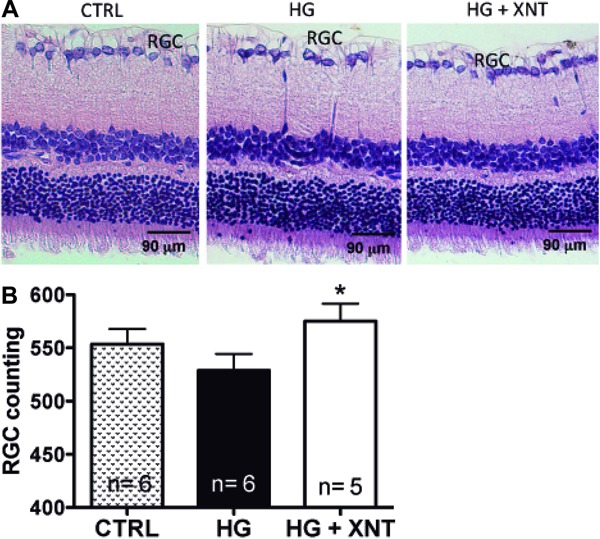
Histological analysis of retinal ganglion cells (RGC). *A*, Representative photomicrographs of retinas showing RGC in control (CTRL), hyperglycemic untreated (HG), and hyperglycemic XNT-treated (HG+XNT) animals. *B*, Quantification of RGC in retinas of rats. Note that the treatment with XNT prevented the loss of these cells in HG+XNT rats. *P<0.05 compared to HG group (one-way ANOVA followed by the Newman-Keuls test).

Hyperglycemia induced slight damage to retinas, as shown by a reduction in the counts of RGC (553.5±14.2 cells in the CTRL group and 530.8±10.3 cells in the HG group, P>0.05); however, this finding was not statistically significant. Chronic administration of XNT preserved the RGC population in retinas of hyperglycemic rats when compared to untreated hyperglycemic animals (530.8±10.3 cells in the HG group and 575.3±16.5 cells in the HG+XNT group, P<0.05, Figure 2), indicating that XNT treatment reduced the cell death of RGC.

### Effects of XNT administration on caspase-3 and VEGF expression

**Figure 3:**
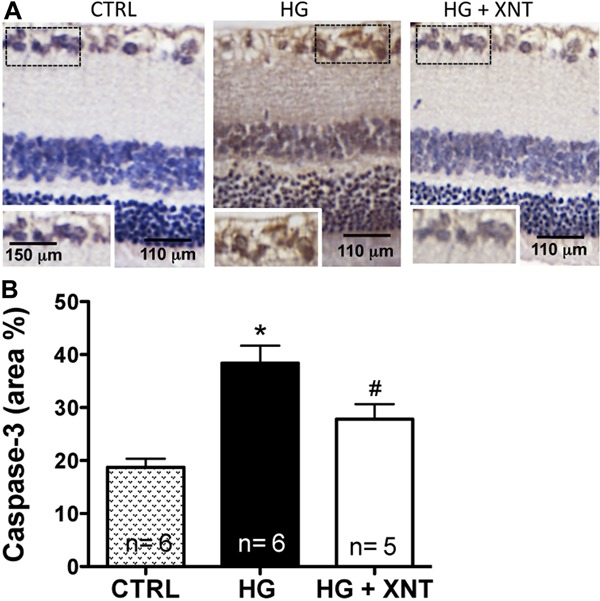
Effects of XNT on caspase-3 expression in retinal ganglion cells (RGC). *A*, Representative photomicrographs of retinas of control rats (CTRL), hyperglycemic untreated rats (HG) and hyperglycemic treated rats (HG+XNT) showing higher caspase-3 expression in RGC of the HG group (see insets). Rectangular dotted lines delineate the amplified areas shown in the inset images. *B*, When the expression of caspase-3 was quantified, XNT reduced the apoptosis of RGC of HG rats. *P<0.05 compared to CTRL group; ^#^P<0.05 compared to HG group (one-way ANOVA followed by the Newman-Keuls test).

To evaluate the mechanisms of action responsible for the protective effects of ACE2 activation on viability of RGC, we analyzed the expression of caspase-3 in these cells. The photomicrographs in Figure 3A show higher caspase-3 expression in the HG group compared with CTRL (CTRL: 18.74±1.59; HG: 38.39±3.39 area%). Treatment with XNT for 30 days was able to reduce the expression of caspase-3 in RGC (38.39±3.39 area% in the HG group and 27.83±2.8 area% in the HG+XNT group, P<0.05, Figure 3B).

**Figure 4:**
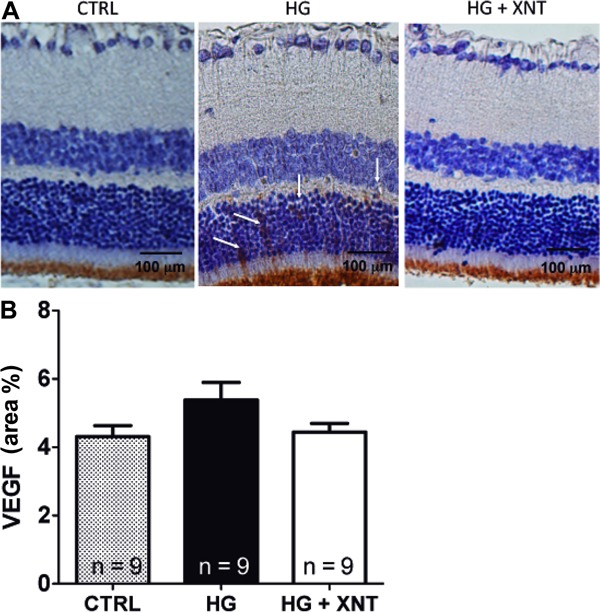
Effects of XNT on vascular endothelial growth factor (VEGF) expression in retinas. *A*, Representative photomicrographs of retinas of control rats (CTRL), hyperglycemic untreated rats (HG), and hyperglycemic treated rats (HG+XNT). The white arrows show VEGF-positive expression. *B*, Quantification of VEGF expression in retinas. No significant differences were observed among the groups (one-way ANOVA).

In addition, the expression of VEGF in retinas of hyperglycemic rats with or without treatment with XNT was evaluated. Although a tendency of decreased VEGF expression was observed in the HG+XNT group compared with HG animals, no significant difference was found between these groups (5.38±0.51 area% in the HG group and 4.31±0.32 area% in the HG+XNT group, P>0.05, Figure 4).

## Discussion

The present study was designed to evaluate the effects of activation of intrinsic ACE2 in retinas of hyperglycemic rats. We found that XNT was effective in preventing early damages to retinas. Specifically, XNT decreased the death of RGC induced by hyperglycemia, probably by reducing the apoptosis of these cells. Overall, our data are consistent with previous studies showing the beneficial effects of ACE2 activation in ocular diseases (13,19,20). For example, Verma and coworkers demonstrated that increased expression of ACE2/Ang-(1-7) in retinas of mice using adeno-associated virus-mediated gene transfer restored the balance of the local RAS (13).

Previous studies have demonstrated the presence of RAS components in eyes (9,12,27-31), including members of the protective branch, i.e., ACE2, Ang-(1-7) and Mas (12,19). In accordance with these data, we observed the presence of ACE2 in retinas of rats, and XNT administration led to an increase in its expression in HG rats. The mechanisms by which XNT augments the expression of ACE2 remain unclear and need more investigation (32). Some possibilities that need to be verified involve the direct action of XNT in ACE2 gene expression or a consequence of a physiological positive feedback mechanism.

Many studies have reported that lesions in neuronal and glial cells may appear early in diabetes (6,7) and that sustained hyperglycemia can induce a progressive loss of retinal neuronal cells (33). Therefore, we quantified the amount of RGC in retinas of hyperglycemic rats with or without treatment with XNT. We found that XNT prevented the decrease in the number of these cells in hyperglycemic animals. This effect was likely due to the reduction in apoptosis, because the expression of caspase-3 was lower in hyperglycemic treated rats. These results corroborate the findings of Oshitari and Roy (34), who found an increased number of apoptotic neurons and an upregulated expression of Bax in retinas of STZ-induced diabetic rats after three weeks of diabetes induction, suggesting that a Bax-dependent pathway is activated in neuronal apoptosis induced by hyperglycemia. In humans, increases in the number of apoptotic cells in retinas were observed in postmortem diabetic patients when compared to nondiabetic controls (35). Among all neuronal cell types present in retina, RGC seem to be the most susceptible to hyperglycemia (36). For instance, Kern and Barber (36) reported a reduction of 23% in RGC in non-obese insulin-deficient mice. Additionally, RGC are highly sensitive to cellular damage and neurotoxicity. VEGF is the most effective angiogenic factor that has been associated with structural and functional alterations in retinas in response to hyperglycemia and/or hypoxia. Thus, this vascular factor has a critical role in the pathogenesis of DR (37,38). Hyperglycemia causes several alterations in capillaries of retinas, including the reduction in nitric oxide levels. Therefore, blood flow in these vessels reduces and a neovascularization process begins, to supply the ischemia. This may explain the high levels of VEGF in ocular fluid of patients with DR (39). In this context, anti-VEGF agents have emerged as a new treatment for diabetic macular edema and retinal neovascularization (40). Consistent with these findings, our results showed a decreased tendency of VEGF expression in retinas of hyperglycemic rats treated with XNT. A significant reduction may be found with a longer time of disease and treatment. Also, the XNT treatment might lead to an improved endothelial function in hyperglycemic treated animals. Indeed, it has been reported that XNT ameliorates the endothelial function of diabetic rats by attenuating oxidative stress (24).

It is important to note that the period of 40 days employed in our protocol is a very short interval to detect the major symptoms of DR, such as capillary damages. However, our main objective in this current study was to investigate the effects of activation of endogenous ACE2 in the early stages of DR. Thus, it is important in future experiments to perform studies using animals with established diabetes for longer periods. Nonetheless, our findings are highly relevant, because even without neovascularization, the main symptom of DR, it was possible to observe neuronal death, which certainly caused loss of visual field function.

In summary, we demonstrated in this study that treatment with the ACE2 activator XNT reduced the apoptotic cell death in retinas of hyperglycemic rats, indicating a role of ACE2 in the pathogenesis of DR.
